# Synchronous Thyrolipoma and Papillary Thyroid Carcinoma: A Rare but Significant Event

**DOI:** 10.3390/diagnostics11081334

**Published:** 2021-07-26

**Authors:** Mariya Kuk, Chu-Jen Kuo, Van-Hung Nguyen, Chien-Chin Chen

**Affiliations:** 1 Department of Pathology, Montreal Children’s Hospital, McGill University Health Centre, Montreal, QC H4A 3J1, Canada; mariya.kuk@mail.mcgill.ca; 2 Department of Pathology, McGill University, Montreal, QC H3A 0G4, Canada; 3 Department of Radiology, Ditmanson Medical Foundation Chia-Yi Christian Hospital, Chiayi 600, Taiwan; Kuo7788@gmail.com; 4 Department of Pathology, Ditmanson Medical Foundation Chia-Yi Christian Hospital, Chiayi 600, Taiwan; 5 Department of Cosmetic Science, Chia Nan University of Pharmacy and Science, Tainan 717, Taiwan

**Keywords:** adenolipoma, cytology, fine needle aspiration, lipoma, papillary thyroid cancer, thyroid, thyrolipoma, thyroidectomy

## Abstract

The presence of adipocytes within thyroid glands is a rare finding seen in thyrolipoma, diffuse lipomatosis, or thyroid teratoma. Although some cases present with multinodular goiter or autoimmune thyroiditis, the exact cause has not yet been elucidated. Among reported cases, thyrolipomas mainly occur in females and usually present as a solitary lesion. However, a few reported cases had coexisting papillary thyroid carcinomas. Herein, we present a 51-year-old female with synchronous thyrolipoma (2.0 × 1.5 × 1.3 cm) and papillary thyroid carcinoma (0.7 × 0.6 × 0.6 cm) within the same thyroid lobe. She had diabetes mellitus and hypertension and complained of anterior neck enlargement and discomfort for three months. Thyroid sonography showed multiple hypoechoic nodules, one of which was heterogeneous and ill-defined. Fine needle aspirate cytology for the ill-defined nodule was suspicious for papillary thyroid carcinoma. She subsequently received radical thyroidectomy and neck lymph node dissection. Histopathologically, one thyrolipoma and one papillary thyroid carcinoma were identified in the right lobe of the thyroid gland without metastases of lymph nodes, while other nodules were multinodular goiter. Notably, thyrolipoma may not be simply an incidental finding but might coexist with thyroid carcinomas. A brief review of the pertinent literature of prior reports is also provided.

## 1. Introduction

A thyroid adenoma abnormally consisting of mature adipose cells is commonly known as thyrolipoma. Although thyrolipoma is a benign entity, rare case reports have mentioned the occurrence of papillary thyroid carcinomas (PTCs) with diffuse thyroid lipomatosis [1] as well as individual thyrolipomas (Table 1) [2,3]. To evaluate a palpable anterior cervical mass on a physical exam, an ultrasound-guided fine needle aspirate (FNA) is a reasonable, cost-effective, and simpler method when compared to computed tomography imaging or surgical investigation [1,2,3,4]. It is important to recognize the existence of lipomas of the anterior neck, usually identified by the presence of only adipose tissue on FNA cytology, which may mimic thyroid nodules on physical examination [4].

Although the FNA samples may be inadequate for diagnosing secondary to scant uninterpretable cellular content, they are usually a suitable first method to assess for malignancy and triage patients concerning the urgency of intervention [6]. Some features that may raise suspicion for malignancy include true papillae, psammoma bodies, and nuclear pseudoinclusions [7]. Based on the features, the FNA sample may be classified according to the 2017 Bethesda system of classification into one of six categories, including nondiagnostic/unsatisfactory, benign, atypia of undetermined significance, follicular neoplasm/suspicious for a follicular neoplasm, suspicious for malignancy, or malignant [7].

Nevertheless, possible differences in malignancy rates between children and adults need to be taken into consideration, especially in cases where the FNA samples are diagnosed as “unsatisfactory”, “atypia of undetermined significance”, or “suspicious for follicular neoplasm”, given that malignancy rates are higher on the resection specimen among children compared to adults within these categories [8].

Surgery is the treatment of choice, especially for neck masses with compressive symptoms [2]. Since thyrolipomas may coexist with PTC or cause compressive symptoms, clinical evaluation and subsequent management are crucial. Herein, we present a rare case of concurrent thyrolipoma and PTC within one thyroid lobe of a diabetic female patient with detailed ultrasound features and a brief literature review. Consent for publication of this case was attained from the patient.

## 2. Case Presentation

**Figure 1:**
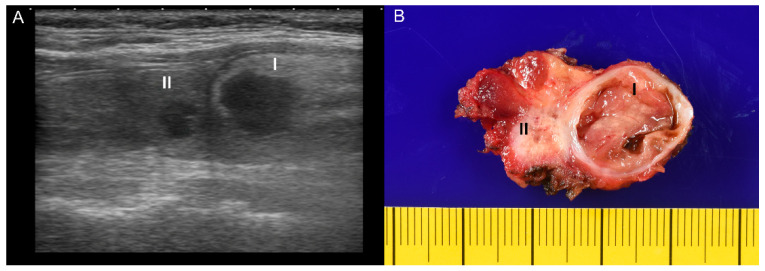
(**A**) The ultrasound examination of both thyroid nodules, referred to as the nodule I (2.3 × 1.9 × 1.4 cm) and the nodule II (1.0 × 1.0 × 0.6 cm), showed hypoechoic and heterogeneous. (**B**) The gross image of the right thyroid from thyroidectomy revealed one well-defined, encapsulated cystic nodule measuring 2.0 × 1.5 × 1.3 cm (I) and one ill-defined, white fibrous nodule measuring 0.7 × 0.6 × 0.6 cm (II).

**Figure 2:**
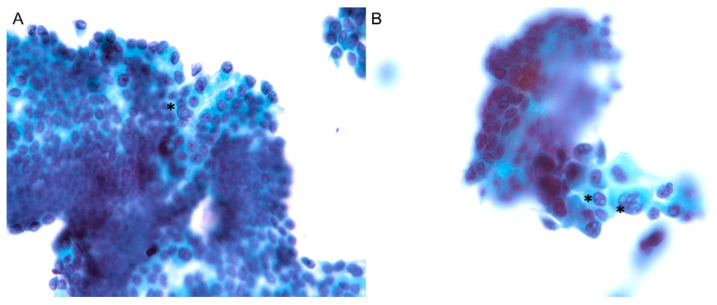
(**A**,**B**). Fine needle aspiration cytology with liquid-based preparation of the nodule II revealed sheets and clusters of atypical follicular cells with nuclear crowding, nuclear grooves, and multiple intranuclear cytoplasmic inclusions (asterisks), highly suspicious for papillary thyroid carcinoma (Papanicolaou stain, ×400).

A 51-year-old female had a medical history of diabetes mellitus type 2 and hypertension under regular medical treatment for more than ten years. She complained of anterior neck enlargement and discomfort for three months. The physical examination showed non-tender, soft, elastic nodularity at the central neck. Ultrasound of the thyroid revealed a total of five hypoechoic nodules in bilateral lobes. Of those, the two largest nodules were in the right lobe, heterogeneous and hypoechoic: (I) well-defined, 2.3 × 1.9 × 1.4 cm, and (II) ill-defined, mildly calcified, 1.0 × 1.0 × 0.6 cm (Figure 1A). An ultrasound-guided FNA was performed on the ill-defined nodule II. The liquid-based cytology showed sheets and clusters of thyroid epithelial cells bearing irregular nuclei, nuclear grooves, and intranuclear cytoplasmic inclusions, which were suspicious for PTC (Figure 2A,B). The patient subsequently underwent radical thyroidectomy and unilateral right neck lymph node dissection. Intraoperative consultation confirmed the nodule II was malignant.

**Figure 3:**
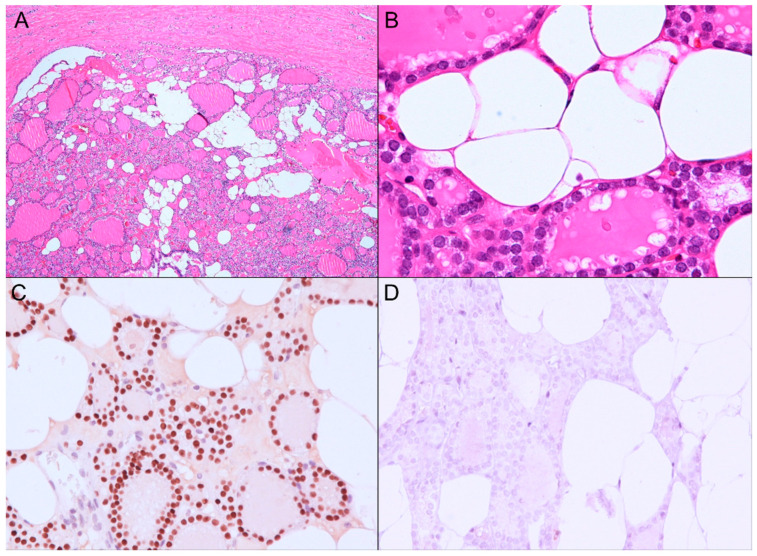
The nodule I: Thyrolipoma. (**A**,**B**) Histopathologically, the nodule I was well encapsulated by fibrous walls and consisted of mature adipocytes and thyroid follicles with a bland-looking morphology. (H&E, A: ×40; B: ×400). (**C**) The thyroid follicular cells were positive for TTF-1 (nuclear positivity, ×200). (**D**) The BRAF staining was negative in thyroid follicular cells (×200).

**Figure 4:**
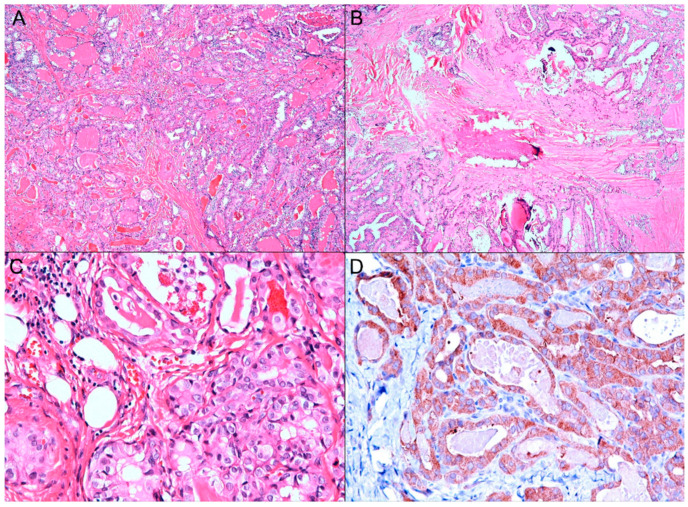
The nodule II: Classical papillary thyroid carcinoma. (**A**) The microscopic examination revealed neoplastic follicular cells arranged as macro or microfollicles with desmoplastic fibrosis (H&E, A: ×40). (**B**) Calcification and stromal fibrosis were seen (H&E, ×40). (**C**) The neoplastic follicular cells displayed nuclear clearing, enlarged nuclei, nuclear grooves, and nuclear inclusions (H&E, ×200). (**D**) The neoplastic follicular cells diffusely expressed BRAF (cytoplasmic positivity, ×200).

In the gross examination (Figure 1B), the right lobe of the thyroid gland revealed two nodules (the larger cystic nodule I: 2.0 × 1.5 × 1.3 cm, and the smaller fibrous nodule II: 0.7 × 0.6 × 0.6 cm), corresponding to the nodules identified on the ultrasound imaging (Figure 1A). On microscopic examination, the nodule I was identified as thyrolipoma containing a variable proportion of mature adipocytes and bland-looking thyroid follicles, which were positive for TTF-1 and negative for BRAF upon immunohistochemistry staining (Figure 3A–D). The nodule II was an unencapsulated classical BRAF-positive PTC (Figure 4A–D). No perineural or lymphovascular invasion was identified, and no extrathyroidal expansion was seen. The left thyroid showed multinodular goiter, and all dissected lymph nodes were negative for malignancy. The patient recovered well and had no recurrence within nine months after surgery.

## 3. Discussion

Identification of adipocytes within a thyroid gland could be classified into six categories based on the lipid content and structure: hamartomatous adiposity, thyrolipoma, fat-containing non-neoplastic conditions, fat-containing thyroid neoplasm, lipid-rich follicular cell lesions, and liposarcoma, as previously described [4].

Although the pathogenesis of diffuse lipomatosis or thyrolipoma is unclear, thyrolipoma may arise as a result of a metaplastic process of stromal fibroblasts secondary to hypoxia or injury [2], or developmental anomalies during embryogenesis and development [2], in which case the lesion would not develop de novo. Of note is that cervical lipomas (relatively common) may be mistaken for thyroid nodules with a differential diagnosis, including thyroid adenolipomatosis, fatty infiltration within amyloid goiter, or thyroidal neoplasms (in particular follicular and papillary neoplasms) [4].

Concurrent presentation of PTC and thyrolipoma is extremely rare and warrants more scrutiny at diagnosis using ultrasound surveillance and FNA sampling. Careful attention must be paid to the classification of the Bethesda system and subsequent management of patients with thyroid nodules within each category, especially given the differences among adults and children [8].

On ultrasound imaging, lipomatous components are usually isoechoic or heterogeneous echogenicity, which may not be easily differentiated from nodular goiter, normal thyroid gland, or parathyroid gland. Notably, in Table 1, the thyrolipomas in published cases measured 2 cm to 15 cm and could be solitary or multiple, which might hinder the interpretation of PTC. The frozen sections of fat-containing lesions may not be well interpreted or well sectioned, thus potentially making correct intraoperative diagnoses more difficult. Therefore, concurrent thyrolipoma and PTC can be a diagnostic challenge due to its rarity, ultrasound characteristics, and potential difficulties in analyzing its frozen sections.

## 4. Conclusions

Adenolipomatosis and thyrolipoma of the thyroid gland are rare yet previously documented phenomena that present either as a solitary entity or in a combination of other thyroid neoplasms or conditions. The current case provides unique images and diagnostic experience of coexisting BRAF-positive classic PTC and thyrolipoma.

## Figures and Tables

**Table 1:** A summary of case reports of thyroid lipomatous lesions coexisting with papillary thyroid carcinoma.

Cases [Reference]	Age, Sex	Size (cm)	Diagnosis	Other Findings
Nandyala et al. [1]	37, M	15 × 11 × 5	Diffuse lipomatosis	PTC, nodular goiter, adenocarcinoma of ascending colon.
Steiner et al. [2]	52, F	4.0 × 3.5 × 2.2	Thyrolipoma	Autoimmune thyroiditis, bilateral PTC, diabetes mellitus, hypertension, hypothyroidism, mass neck compression.
Cameselle-Teijeiro et al. [3]	28, F	Multiple small nodules.	Thyrolipoma	Lymphocytic thyroiditis, multinodular goiter, PTC (follicular variant), lipoma of neck, Cowden syndrome.
Laforga et al. [5]	58, F	3.8	Thyrolipoma	PTC
Current case	51, F	2.0 × 1.5 × 1.3	Thyrolipoma	PTC, diabetes mellitus, hypertension.

M: Male; F: Female; PTC: Papillary thyroid carcinoma.

## Data Availability

Not applicable.
